# E-cigarettes and youth: an unresolved Public Health concern

**DOI:** 10.1186/s13052-022-01286-7

**Published:** 2022-06-14

**Authors:** Fabrizio Virgili, Raffaella Nenna, Shira Ben David, Enrica Mancino, Greta Di Mattia, Luigi Matera, Laura Petrarca, Fabio Midulla

**Affiliations:** Department of Maternal Infantile and Urological Sciences, Sapienza University of Rome, Viale Regina Elena 324, 00161 Rome, Italy

**Keywords:** Adverse effects, Children, Electronic cigarette, Pediatric, Public Health, Vaping

## Abstract

The use of electronic cigarettes (e-cigarette) and vaping devices started as a potential aid for cessation and reducing the harmful consequences of cigarette smoking, mainly in the adult population. Today e-cigarette use is highly increasing in vulnerable populations, especially young and pregnant women, due to the misconception of its harmless use.

Despite the growing acknowledgment in e-cigarette as a potential harmful device, and due to mixed information found concerning its beneficial aid for smokers, along with an insufficient clinical study done in human models, it is important to further evaluate the possible benefits and risks of non-combusting, vaping nicotine or non-nicotine delivery devices.

In this review we tried to summarize the latest updated information found in the literature, concentrating mainly in the variety of adverse effects of e-cigarette use and its contribution for recent and future health concerns.

## Introduction

Tobacco smoke undoubtedly remains world’s leading cause of preventable disease [1]. Over the last decade, reducing cigarette consumption has become a Public Health goal, therefore prevention campaigns intensified and restrictions on their marketing and access escalated [2].

Electronic cigarettes (also known as E-cigs, Electronic Nicotine Delivery Systems-ENDS, vaping device or e-vaporizers) are an electronic device that can vary in size and shape, consisting of a battery, an electrical heater and a liquid, which is aerosolized to be inhaled. Liquid composition can include nicotine, a solvent and an utmost variety of flavorants [3]. Taking shape as a smoking cessation strategy or – in actual fact – as a legal alternative where conventional smoking was prohibited, e-cigarettes, invented by Hon Lik (a Chinese pharmacist), were patented in 2003. However, only in 2007 they became commercially available in USA and Europe [4].

In contrast with a consistent decline in smoking prevalence among youth [5], over the past few years electronic cigarettes have rapidly gained popularity to the point of becoming the most common tobacco product in this age group [6]. Their social acceptance, together with their widespread availability, contributed to drastically increase primary use by adolescents and second-hand exposure in children, outlining the need for an assessment of their health effects in these categories [7].

In 2018, the National Youth Tobacco Survey reported that 20.8% of high school students and 4.9% of middle school students currently used e-cigarettes [8]. Since the introduction of pod-based devices, vaping prevalence has tremendously increased, reaching 28% in 2019 [9] and even 40.5% among 12th graders [10]. In Great Britain, during 2020 16.4% of 11–18-year-old students had tried (at least once) e-cigs, compared to 15.4% in 2019 and to 12.7% in 2015. Also current use increased since 2015 from 2.4 to 4.8% [11]. Prevalence of current e-cigarette smokers in Italy doubled from 2014 (8%) to 2018 (18%), whereas the number of ever smokers has risen by 60% (from 28 to 44%) [12]. According to forecasts, e-cigarettes sales will surpass those of traditional tobacco by 2023 [13].

Marketing has certainly played a major role in vaping prevalence inflection among children and adolescents. E-cigarettes can be purchased in vape shops, tobacco vendors, gas stations, groceries, pharmacies and even online [14]. The manufacturing companies, often owned by tobacco firms, address their products to youth by promoting appealing flavours and using multiple communication channels: television advertisings; targeted advertisements at the point of sale; web sites and social media; celebrity partnerships; free samples at youth-oriented events [15–17]. In 2016, 78.2% of middle and high school students have been exposed to e-cigarette advertisements from at least 1 source and increasing exposure seemed associated with higher odds of use [17]. Social media are easily accessible by teenagers and convey the use of e-cigarettes as socially acceptable [18]; despite ENDS being born as a smoking cessation strategy, less than 1% of twitter posts concerning vaping are related to smoke cessation [19]. Apparently, only 8% of adolescents take up e-smoking as a nicotine replacement strategy [20] whereas the most common reasons underlying vaping experimentation in pediatric population are: curiosity, social influence, availability at low cost, enjoyable flavors, ease of concealment [21–23]. Sustained use is then encouraged by misperceptions about safety, nicotine content and social prevalence [21, 24–26].

A cross-sectional analysis pointed out that positive expectancies regarding e-cigarette use (e.g. gaining respect of peers and chances of being liked by partners, reducing stress, enjoying throat sensation) are related with a greater prevalence of current use [27]. Users exhibit the lowest perceptions of harm and more positive attitudes towards e-cigarettes when compared with non-users [28]. Adolescents perceptions – which affect their decision-making process – are generally biased in the direction of their own experience, a phenomenon referred to as “false consensus effect”[29]: for instance, teens tend to overestimate actual smoking rate among peers [30], therefore they may be more prone to develop such addiction. In this regard, Gorukanti et al. [28] administered 9th and 12th graders from California an online survey to investigate their attitudes towards e-cigarettes and whether they differ by past use. Findings showed that prevalence of both e-cigarette and cigarette use among parents, siblings, and close friends was higher in adolescents who have ever used an e-cigarette. At the same time, vapers believed more peers and relatives smoke e-cigarettes than do non-users. A worrying percentage of participants, especially smokers, thought that e-cigs contained water and claimed that vaping felt cleaner and safer than traditional smoking. Nonusers, instead, were more prone to consider e-cigarette harmful to children.

### Device evolvement

Throughout the years, e-cigarettes design and technical features evolved, providing the market with updated products meeting consumers’ different demands. E-cigarette’s modernisation process also helped contain health implications. First generation ECs, usually referred to as cig-a-likes, have been conceived to resemble the design and feeling of traditional cigarettes. In early models, friable solder joints could release in aerosols particles of tin (associated with stannosis and pneumoconiosis [30]), a flaw remedied by coating the thick wire with silver, using rigid joints external to the atomizer or connecting wires without solders rather through clamps [31, 32]. The thick nickel or copper wire, tin- or silver-coated, was not included in second generation products and following [33]. By removing the silicon cover from later products, its presence in aerosols drastically decreased [34]. Nonetheless, the empowerment in second and third generation batteries, along with bigger atomizers and higher amounts of metal, allowed to generate larger volumes of aerosol [35], resulting at the same time in a greater transfer of particles, metals, toxicants [36, 37]. Furthermore, as voltage/power ratio increased, new potentially toxic by-products could emerge from the liquid [34]. Likewise, in larger reservoirs such as those of second and third generation ECs, fluid stagnation could enrich aerosols with additional toxicants through repeated use [37]. Fourth generation ECs, referred to as pod mod devices, have become popular among teenagers as a socially acceptable alternative to conventional cigarettes due to their stylish design (e.g. USB or teardrop shape), wide selection of flavours and user-friendly functions [38, 39]. Their likeness to an USB memory stick allows them to be discretely used in no smoking areas and easily concealed from parents, contributing to a new widespread phenomenon, known as “stealth vaping” [19, 40, 41]. A distinctive feature of fourth generations devices is the use of nicotine in its protonated form, which reduces the irritating effect on throat mucosa while increasing the amount of nicotine delivered in aerosols [42]. The heterogeneity outlined above complicates research on potential health effects, since the variability in design and technical features prevents us from discussing e-cigarettes as a single device. Furthermore, besides type and age of the device, e-cigarette health impact depends on multiple variables including ambient factors (e.g. climate conditions, room size and density of people) and user’s habits (puff length and frequency) [43].

### Content

Aerosol composition is affected even by the solvent employed, being vegetable glycerin (VG), propylene glycol (PG) and their mixture the most widely used. They can also influence particle-size distribution, therefore the region of deposition in the respiratory system [44]. A higher percentage of propylene glycol seems enhancing flavour and strengthening the so-called “throat hit”, whereas a higher percentage of vegetable glycerine may increase vapor production [45]. Vegetable glycerine exposure has been associated with irritation of eyes, lungs, and oesophagus mucosa [46]. Likewise, its higher boiling point requires the heating element to reach higher temperatures, resulting in a greater risk of toxicants emission [47]. The highest yield of aldehydes occurs in devices containing propylene glycol [48], also related to upper respiratory infection-like symptoms [49].

A vast amount of studies aimed at characterizing e-cigarettes emissions and variously reported measurable amounts of ethanol, volatile organic compounds (VOCs), polycyclic aromatic hydrocarbons (PAHs), silicon, lead, nickel, air pollutant, formaldehyde, acetaldehyde, isoprene, acetic acid, 2-butanodione, acetone, and propanol [50–52]. Volatile organic compounds can provoke eye and respiratory tract irritation, neurological impairment and liver damage [53]. Polycyclic aromatic hydrocarbons demonstrated carcinogenic, respiratory, immunological, neurological and reproductive effects [54]. Reactive carbonyls such as aldehydes and acrolein (product of glycerol constituents vaporization [55]) elicit airway constriction, direct damage to airway epithelium and alterations in gene expression, in addition to neutrophils activation, degranulation and apoptosis [56].

The extraordinarily wide variety of flavorants available amplifies the heterogeneity in E-cigs aerosol composition. These chemical components are generally employed in food industry and recognized as safe additives but this does not imply their harmlessness when inhaled [57]. Some are known allergens (e.g. cinnamaldehyde for cinnamon aroma) [58], others may provoke ocular and airway irritation (e.g. benzaldehyde for fruity aromas) [59]. Pre-clinical studies demonstrated that flavoring chemicals elicit pro-inflammatory responses in lung epithelial cells and fibroblasts and decrease transepithelial resistance in bronchial epithelial cells [60]. Likewise, diacetyl and acetyl propionyl (butter flavoring volatiles) seem to underlie bronchiolitis obliterans, as seen in microwave pop-corn producing factory workers [61, 62]. Besides, flavored e-cigarettes are misleadingly considered less harmful than those with tobacco flavor, therefore used carelessly by youth [63].

An additional concern is represented by the inconsistencies between declared nicotine levels and actual nicotine content, which have been detected even in liquids supplied from the same company [64].

Moreover, e-cigs nicotine derives from tobacco plant, thus e-liquid can include other tobacco-related toxicants, such as tobacco-specific nitrosamines (TSNAs) [65].

### Vaping health implications

An uncountable amount of studies aimed at evaluating e-cigarette potential consequences on human health and the most updated panorama of scientific literature provides increasing evidence of vaping harmfulness. According to the centre for disease control and prevention (CDC) [66], although e-cigarettes harmful effects are fewer in comparison to burned cigarettes, they are considered unsafe, harmful for brain development and increase the risk to future addictions especially in kids, adolescence and young adults.

#### Respiratory effects

Among e-cigarettes adverse health effects, respiratory impact is by far the most extensively studied. In the same way as conventional cigarettes smokers, vapers’ pulmonary epithelium is typically damaged [67] and bronchial mucosa chronically inflamed [68]. Proteomics of e-cigarette users’ sputum document higher levels of myeloperoxidase, neutrophil elastase and proteinase-3, indicative of neutrophil activation. Furthermore, chronic vapers’ neutrophils display a greater propensity for NET formation when compared to cigarette smokers or non-smokers [56]. The significant decrease in Forced Expiratory Volume in 1 s and fractional exhaled Nitrogen Oxide, both observed in e-cigarette users, are suggestive of an underlying bronchial inflammation [68–70].

From a clinical perspective, these pathophysiological alterations could underlie the considerable increase in asthma and bronchitic symptoms reported by e-cigarettes users, especially adolescents. According to various studies involving high school students, vapers have a twofold higher risk of chronic cough, phlegm or dyspnea, together with a greater incidence of asthma [71, 72]. A higher prevalence of e-cigarette use is reported among adults living with a child affected by asthma [73], whose risk of acute exacerbation can increase by 30% [74]. Schoolwork is indirectly affected too, as a result of absenteeism secondary to the aforementioned symptoms [75]. Preclinical studies [76] suggest also a detrimental effect on mucociliary clearance which, coupled with a decreased cough sensitivity [77] and the overexpression of PAF-R (pneumococci’s receptor [78]), predispose vapers to increased rates of pneumonia [79].

In parallel to the increasingly wide distribution of e-cigarette, a growing number of cases helped characterize a new nosological entity, which is now referred to as E-cigarette or Vaping Associated Lung Injury (EVALI). It is a diagnosis of exclusion that requires use of e-cigarettes and related products within the previous 90 days, in addition to pulmonary infiltrates on imaging [80]. The prevalence seems higher in youth: indeed, the median age of the initially reported cases was 19 [81]. The hypothesized causative agent of lung injury is vitamin E acetate, which may be found in cartridges of THC flavored e-cigarettes, widespread among high school students [82]. Its aerosolization generates ketene, that is irritant to airways and disrupts phospholipid bilayer decreasing surfactant effectiveness [83]. EVALI can occur with shortness of breath, cough, tachycardia, tachypnea, pleuritic pain and rarely hemoptysis. Nausea and abdominal pain, as well as fevers and chills are not infrequent. Up to 30% of the affected require mechanical ventilation and up to 70% the admission to intensive care units [83–85]. Bilateral lower-lobe predominant ground glass and consolidative opacities with subpleural sparing are the most typical findings at chest imaging. Other possible radiographic patterns include multiple dense consolidations (as in Acute Respiratory Distress Syndrome), multifocal patchy opacities (as in Cryptogenic Organizing Pneumonia) or ground glass pattern with air trapping (as in hypersensitivity pneumonitis) [85, 86]. A bronchoscopy with Broncho-Alveolar Lavage and, if possible, transbronchial biopsies should be performed after ruling out cardiac causes (through chest X-rays and/or echocardiography) and other respiratory or systemic infections (viral assay, urine, sputum and blood cultures), consistently with clinical severity [83, 87]. Steroids should be started concomitantly with antibiotics, especially in patients with respiratory failure; in less severe presentations they can be delayed after infectious causes are ruled out. Response to methylprednisolone is generally excellent [84]. Based on the severity of the clinical picture, patients can benefit from high-flow oxygen therapy, noninvasive ventilation or require mechanical ventilation [81, 84, 88].

Case reports point out an association between e-cigarette smoking and lipoid pneumonia, acute eosinophilic pneumonia, subacute bronchial toxicity and even reversible cerebral vasoconstriction syndrome [89–92].

#### Cardiovascular effects

Laboratory studies have pointed out that exposure to e-cigarette can induce platelet aggregation by upregulating expression of CD41 (GPIIb), CD42b, and CD62p (P-selectin) [93]. It also underlies oxidative stress elevation, impairment of antioxidant defenses (vitamin E reduction) and endothelium dysfunction/damage, evidenced by the detection of endothelial progenitor cells and microvescicles into the bloodstream [94–96]. All these alterations could play a role in cardiovascular risk increasement. It has been proven, indeed, that daily vaping represents an independent risk factor for myocardial infarction [97], synergically amplified by exposure to nicotine, a parasympathomimetic alkaloid increasing heart rate and blood pressure [98]. Additionally, a 30 minutes vaping session seems inducing an unfavorable effect on aortic stiffness similar to traditional smoking [99].

#### Neurological effects

Nicotine is widely recognized as a psychoactive substance [100]. Vapers experience craving, impaired capacity to stop and withdrawal symptoms during abstinence (e.g. irritability), which all suggest e-cigs potential of inducing nicotine dependence [101, 102]. Similarly to other substance use disorders, adolescents are characteristically more vulnerable to addiction [103].

Despite ENDS representing a tobacco cessation strategy, the risk of transition to conventional cigarettes in previously never smokers represents an emerging issue of critical relevance, especially in young people [104–107]. A recent meta-analysis showed that in a population of teens and young adults who have never smoked, odds of smoking initiation were 3 to 6 times higher in those who have ever used e-cigarettes [108]. Evidence suggest also that elevated nicotine concentrations may heighten the likelihood of progression [109]. In this perspective, the renormalization of a smoking culture among teenagers threatens to subvert decades of anti-smoking efforts.

Structural and neurochemical changes in the central nervous system lie beneath the behavioral evolution that characterizes adolescence. Against this background, nicotine can affect its regular course, contributing to attention and cognitive deficits and exacerbating mood disorders [110].

Furthermore, in such a critical phase of human development, nicotine exposure may prime the brain’s reward system increasing pleasing effects of other substances of abuse [111, 112]. As evidence of this, youngsters smoking e-cigarettes display a greater risk of co-occurring alcohol and/or marijuana use [113].

#### Gastroenteric effects

In spite of small evidence on the matter, e-cigarettes’ detrimental effects do not spare gastrointestinal system. Smoking represents a well-known risk factor for gastro-esophageal reflux, especially since nicotine has been shown to regulate transient lower esophageal sphincter relaxations [114]. Electronic nicotine delivery systems have been associated to esophageal symptoms and are now considered potential triggers of esophagitis exacerbations [115]. Animal studies pointed out that vaping can induce hepatic steatosis through heterogeneous mechanisms, involving oxidative stress, hepatocytes apoptosis and impairment of cholesterol and lipid metabolism [116]. A recent case report documented e-cigs-induced hepatic injury even in humans, describing an increase of liver enzymes in a young vaper presenting to the emergency department for fever, abdominal pain, vomiting and diarrhea [117]. In this regard, clinicians must always consider – or better solicit – patient’s vaping history, since health effects of electronic cigarettes are disparate and sometimes unexpected.

#### Acute adverse effects

As regards e-cigarettes potential harmful effects, consideration should be given to acute injuries. A fair amount of reports to poison centers concern children incidentally exposed to e-cig liquids through ingestion (70%), inhalation (15%), ocular (8.5%) and dermal contact (6%) [118]. Nicotine poisoning can result in tachycardia, dizziness and even seizures [119]. Ingestion of 0,1 mg/kg of nicotine-containing fluid can be fatal to a child [7]. Acute exposure to e-cigarettes seems associated with a worse prognosis compared with that of conventional tobacco [120]. As electronic devices, batteries could also explode provoking severe burns [121].

#### Second- and thirdhand exposure

Concerning e-cigarettes impact on pediatric population, an often-underestimated aspect is second- and third-hand smoke. Approximately 20% of parents using e-cigarettes follow strictly enforced vape-free home and car policies; among dual users, 64% has a smoke-free and only 26% a vape-free home policy, which implies a general misperception about e-cigarettes safety, especially among younger parents [122]. There is evidence that nicotine metabolites in serum, as well as saliva and urine cotinine levels, are superimposable among non-using adults secondhandedly exposed to e-cigarettes and conventional cigarettes [123]. Even secondhand particular matter exposure levels can equal that of traditional smoking [124] and appear to be greater in nicotine-free devices [125].

#### Vaping during pregnancy (fetal developmental effects)

Of no less importance is fetal exposure to nicotine during pregnancy. Vaping prevalence in pregnant women has been estimated to stand between 0.6 and 15% [126]. Nicotine can cross the placenta and measurable nicotine levels can been detected in offspring of mothers smoking during pregnancy [127]. In uterus nicotine exposure increases the risk for eclampsia, premature birth, cleft lip and palate, reduced birth weight [128–130], sudden infant death syndrome, altered corpus callosum, auditory defects, besides being related to future compromised fertility, type 2 diabetes, obesity, hypertension and respiratory dysfunction [131, 132]. Nicotinic acetylcholine receptors regulate critical stages of brain development and nicotine neurotoxic effects on the developing brain have been widely demonstrated, including future hyperactivity, cognitive impairment, anxiety, mood and attention symptoms, sensitivity to stimulant drugs [131, 133–137]. To date, e-cigarette impact on pregnant women and fetuses remains uncertain and further research should be established. A vast amount of studies carried out on animal models suggest pre- and postnatal alterations related with the exposure to both, nicotine and nicotine-free aerosols, including the down-regulation of genes implied in lung development [138], an inverse relationship between plasma and urine cotinine level and body weight [139], in addition to neuro-behavioural and developmental disorders similar to those resulting from conventional cigarette exposure [140].

#### E-cigarettes and COVID-19

In the course of COVID-19 pandemic, forced social isolation due to quarantine measures has been associated with the onset and exacerbation of psychological problems, including substance use [141]. Boredom, loneliness, and loss of daily structure during confinement led people to consume larger quantities of alcohol and smoke more cigarettes, an ever-worrying global concern [142]. In Italy, e-cigarette use increased by 12.1% and almost 2% of non-users started vaping during the lockdown, especially vulnerable categories such as youngsters, drug-addicted (of less available substances) or people experiencing anxiety symptoms (worsened by the pandemic background) [143]. In such a dramatic framework, e-cigarette companies seized the moment to exploit a global pandemic for their marketing purposes: using tv advertising and social media (hence addressing to younger customers) they offered pandemic supplies (e.g., masks, hand sanitisers) as gifts with the purchase of their products, suggested online shopping with pandemic-themed discount codes (e.g. “STAYHOME”) and encouraged home vaping to cope with confinement-related stress [144].

Most concerning is that smoking and vaping has proven to impact on individual’s susceptibility to coronavirus infection, rate of symptomatic disease course other than overall prognosis [145–147]. E-cigs users display indeed a 5–7 times higher risk of COVID-19 diagnosis compared to non-users [145]. Mask removal and the gesture of vaping facilitate viral hand-to-mouth transmission (especially with shared devices), increasing smokers’ susceptibility to infections, which is synergically enhanced through lung inflammation, impaired clearance, oxidative stress and immune dysregulation [147]. Angiotensin-converting enzyme 2 (ACE-2), a ubiquitous transmembrane metallocarboxypeptidase, is the receptor SARS-CoV2 utilizes to enter human cells [147]. Recent research provides evidence that a nicotine-related overexpression of ACE-2 in bronchial epithelial cells is mediated by α7-subtype nicotinic receptors (nAChRα7), which could facilitate SARS-CoV2 biological cycle [148, 149]. Furthermore, chronic exposure to nicotine aerosol seems responsible for lung inflammation through a dysregulated repair response and extracellular matrix remodelling mediated by nAChRα7 itself [150]. In this regard, ECs may not only ease virus entry, but even exacerbate the resulting lung injury. E-cigarettes detrimental effects are not confined to respiratory involvement: vaping and its consequent low-grade brain inflammation results in a procoagulant condition increasing stroke risk, a known and fearsome complication of SARS-CoV2 infection [151]. Although direct evidence remains forthcoming, such mechanisms are supposed to be mediated by ACE-2 pro-inflammatory pathway, which is extensively expressed in central nervous system too [147]. Electronic smoking has also been proven to adversely affect the clinical course of SARS-CoV2 infection: McFadden et al. recorded significantly higher rates of COVID-19 symptoms among vapers when compared to non-users, with manifestations of increased severity in dual users (traditional and e-cigarettes) [152]. Within the framework of the ongoing COVID-19 pandemic, such findings oriented the scientific interest towards the relationship between SARS-CoV2 and exposure to nicotine, yet current literature provides inconsistent conclusions. Cross-sectional data from HEBECO study excluded any association between COVID-19 diagnosis and vaping habits [153], while Miyara and colleagues have even documented a lower probability of a symptomatic or severe disease in current smokers as compared to the general population [154]. This led to hypothesize that nicotine might achieve a preventive effect. nAChRα7s are known to modulate the phlogistic response by calcium-mediated activation under inflammatory conditions (such as cytokine storm in severe COVID-19) thus some Authors speculate that nicotinic agents might represent a therapeutic opportunity for acute COVID-19 [155, 156]. In the light of available evidence, we can presume that nicotine antiphlogistic effect (potentially achieved through nAChRα7 modulation) mitigates the inflammatory response to the virus and acute lung injury, but chronic exposure may possibly increase the susceptibility to its infection [147].

Interestingly, SARS-CoV2 respiratory involvement is associated with an increase of CCL2–4 levels in bronchoalveolar fluid and mononuclear cells, an alteration recorded also in EVALI, whose clinical and radiological presentation is to a certain extent similar [157–159]. Chest imaging can occasionally help narrow the diagnosis since COVID-19 frequently presents with multifocal ground-glass peripheral opacities, on the contrary subpleural sparing is common in vaping-associated lung injury [160]. Despite the absence of pathognomonic features, EVALI should be considered in younger patients, displaying leucocytosis and responsive to corticosteroids (being COVID-19 more likely in older ages and often associated with lymphopenia) [159]. Accordingly, vaping history should always be investigated – and excluded – in young patients presenting to emergency departments for acute fever and respiratory symptoms, especially in individuals with no epidemiologic links to SARS-CoV2 infection [158, 161].

## Conclusions

Traditional tobacco impairing effects have been widely demonstrated, in contrast, vaping mid- and long-term complications remain unclear. Nonetheless, there is increasing evidence that e-cigarettes can no longer be considered as harmless devices. As a result of vaping cross-generational diffusion, e-cigarettes health implications have become an issue of pediatric interest since children are at greater risk of their chronic effects. Despite its lesser harmfulness than traditional smoking, a vast amount of research revealed that vaping is far from being safe. Its risk-benefit ratio remains acceptable only if used as a smoking-cessation device, available under medical prescription for teenagers truly planning to quit traditional smoking for good. The remaining pediatric population does not get any benefit from electronic smoking. Therefore, based on a precautionary principle e-cigarettes should in any event be considered unhealthy hence consequently banned in this age group and restricted in its close contacts. In this perspective, governments should strengthen prevention strategies as well as restrictions and regulations on e-cigarettes marketing and advertisings. In parallel, teachers and pediatricians must play a crucial role in children and parents’ education by raising their awareness about vaping harmful effects and dispelling widespread misperceptions. Given its ever-growing relevance as a worldwide health concern, further investigation in this regard is in our main interest.

## Data Availability

N/A
